# Validating the Dutch Eating Behavior Questionnaire for Children (DEBQ-C) among Korean children and adolescents with high weight

**DOI:** 10.1186/s40337-023-00894-w

**Published:** 2023-10-06

**Authors:** Na Young Kim, Sooyeon Suh, Jieun Kim, Kumhee Son, Sarah Woo, Jia Kim, Kyung Hee Park, Hyunjung Lim

**Affiliations:** 1 Department of Medical Nutrition, Graduate School of East-West Medical Science, Kyung Hee University, Yongin, 17104 Korea; 2 Department of Psychology, Sungshin Women’s University, Seoul, 02844 Korea; 3 Research Institute of Medical Nutrition, Kyung Hee University, Seoul, 02447 Korea; 4 Department of Medical Sciences, College of Medicine, Hallym University, Chuncheon-Si, 24252 Korea; 5 Department of Clinical Psychology, National Mental Health Center, Seoul, 04933 Korea; 6 Department of Family Medicine, Hallym University Sacred Heart Hospital, Anyang, 14068 Korea

**Keywords:** Dutch Eating Behavior Questionnaire-Children (DEBQ-C), Overeating, Validity, Children, Adolescents, Obesity

## Abstract

**Background:**

Using reliable measurement tools is becoming increasingly important as the prevalence of obesity among children increases in Korea. The Dutch Eating Behavior Questionnaire for Children (DEBQ-C) measures three eating behaviors associated with overeating. This study aims to validate the DEBQ-C for use among Korean children and adolescents with high body weight. It examines the psychometric features of the Korean translation of the DEBQ-C and investigates the relationship between the subscale scores of the DEBQ-C and the weight status of participants (categorized into *overweight*, *obese*, and *morbidly obese*).

**Methods:**

A total of 233 children and adolescents (mean age: 11.4 ± 1.6 years) completed the questionnaire. The study verified the factor structure of the DEBQ-C using confirmatory factor analysis (CFA) and estimated its internal consistency with Cronbach’s alpha. For convergent validity, it employed Pearson’s correlation coefficient to assess relationships between the three eating behaviors of the DEBQ-C and the number of food addiction symptoms of the Yale Food Addiction Scale for Children (YFAS-C). Lastly, it examined the relationship between DEBQ-C scores and weight status via multinomial logistic regression analysis.

**Results:**

The three-factor model demonstrated goodness-of-fit (χ^2^ = 253, *df* = 167, χ^2^/*df* = 1.515, *p* < 0.001, CFI = 0.944; TLI = 0.937; RMSEA = 0.047). The internal consistency of the three eating behaviors was also satisfactory (Cronbach’s alpha = 0.707–0.890). The emotional and external eating subscales of the DEBQ-C were positively correlated with the number of symptoms of food addiction of the YFAS-C. Emotional (OR: 2.008; 95% CI 1.973–2.043) and external (OR: 2.074; 95% CI 2.029–2.119) eating were positively associated with obesity status.

**Conclusion:**

The results suggest that the Korean version of the DEBQ-C is suitable for the examination of problematic eating behaviors in Korean children and adolescents with high body weight.

## Abstract (plain language summary)

The proportion of children and adolescents with obesity in Korea is increasing. Thus, the availability of a tool that can reliably identify problematic eating behavior linked to obesity would assist with prevention and management efforts. The Dutch Eating Behavior Questionnaire for Children (DEBQ-C) measures three types of overeating behavior in children that influence weight gain. This study examines the suitability of the Korean translation of the DEBQ-C questionnaire for use in studies on Korean children and adolescents with high body weight. The results of a survey on 233 adolescents (average age: 11.4 ± 1.6 years) suggest that the DEBQ-C translation would be useful in investigating overeating behavior in Korean children and adolescents. In addition, the findings suggest that eating in response to emotions and external cues are likely to play a role in the development of obesity among children and adolescents.

## Introduction

The prevalence of obesity among children and adolescents is increasing worldwide [1]. According to the 2019 Student Health Examination Sample Survey, 25.8% of Korean children or adolescents were overweight or obese, and this proportion has been increasing every year over the past five years [2]. Additionally, the proportion of children and adolescents with morbid obesity has been steadily increasing from 0.8% in 2008 to 2.0% in 2017 [3]. These rates are alarming due to the strong evidence that obesity in childhood and adolescence poses a high risk of progression into adult obesity, which subsequently increases the risk of chronic diseases such as diabetes and cardiovascular disease (e.g., hypertriglyceridemia, hypercholesterolemia, and high blood pressure). In addition, children and adolescents with obesity may experience low self-esteem, depression, and social phobia, and other impacts on social and psychological development [4]. This scenario indicates the importance of managing and preventing obesity during childhood and adolescence.

Diverse variables, such as dietary intake, physical activity, sedentary behavior [5], and genetic background [6], influence childhood obesity. However, the most proximal cause is an energy imbalance between calories consumed and expended [7]. Excessive energy intake can be a result of problematic eating behaviors influenced by internal and external factors such as an individual’s emotional state and environmental cues [8]. Therefore, identifying the factors that influence excessive energy intake is necessary. Moreover, recognizing and improving problematic eating behaviors help prevent and overcome obesity [9], which calls for the need for validated questionnaires that can assess problematic eating behaviors.

The Dutch Eating Behavior Questionnaire for Children (DEBQ-C) [10] assesses three eating behaviors that potentially contribute to weight gain. The DEBQ-C is a pediatric version of the DEBQ [11], and the latter is widely used in the research on eating habits. The DEBQ-C consists of three subscales that explore the important aspects of problematic eating, namely, emotional eating (food is consumed due to negative emotional states) [12, 13], external eating (food is consumed due to external circumstances regardless of internal hunger) [14], and restrained eating (calorie intake is restricted to lose weight). Restraint theory assumes that chronic caloric restriction as a method for losing weight is unsustainable for many individuals and can eventually lead to disinhibited eating triggered by external or internal stimuli [15].

The DEBQ-C has been validated in Taiwan (age: fifth- and sixth-graders in elementary school; weight: normal, healthy, overweight, and obese) [16], Spain (age: 10–14 years; weight: normal, overweight and in treatment, and overweight not in treatment) [17], and Japan (age: fifth- and sixth-graders in elementary school and first- and second-graders in junior high school; weight: underweight, normal weight, and overweight) [18]. All studies have reported a three-factor structure. Thus, the validity of the questionnaire for use on children and adolescents has been confirmed in clinical and nonclinical samples.

With the increased prevalence of overweight and obesity among children in Korea, the availability of reliable and effective tools that can be used to better understand overeating behavior in the Korean population is important. The main objective of the present study was to confirm the validity of the Korean version of the DEBQ-C when used in studies on Korean children and adolescents with high body weight. The secondary objective was to examine the relationship between the three eating dimensions of the DEBQ-C and varying degrees of overweight.

## Methods

### Participants and procedure

This study applied a cross-sectional design on a total of 233 participants (age range: 7–17 years). The participants were recruited through school flyers and the official website of Seoul. The inclusion criteria were (a) overweight children and adolescents with age- and sex-specific BMI ≥ 85th percentile according to the 2017 Korean National Growth Charts and (b) living in Seoul, Gyeonggi, and other cities (e.g., Chungcheong-do, Incheon-si, and Bucheon-si). The participants were excluded if they were prescribed medications that can influence weight status, including corticosteroids, thyroid hormones, and insulin.

Consent was obtained from both the participants and their parents or caregivers. The study received ethics approval from the Institutional Review Board of Hallym University Sacred Heart Hospital (IRB No. 2019-04-027-003).

### Anthropometric measurement

To obtain anthropometric data, an experienced staff member measured weight, height, waist circumference, and hip circumference. We classified the weight status of the participants according to the 2017 Korean National Growth Charts for children and adolescents and based on age and sex (overweight: BMI in the 85th–95th percentile; obese: BMI ≥ 95th to 120th percentile; morbidly obese: BMI ≥ 120th percentile). We calculated the *z*-scores for BMI using the LMS method based on the 2017 Korean National Growth Charts for children and adolescents.

### Translation and Back-translation of the Dutch Eating Behavior Questionnaire for Children (DEBQ-C)

The DEBQ-C underwent a translation–back-translation procedure [19]. The original version of DEBQ-C was translated into Korean, and the first translation version was translated back into English. Specifically, as the first step, a bilingual researcher and a person with a Ph.D. in medical nutrition translated the original version into Korean. In the second stage, a psychology professor who was fluent in English and Korean and who did not participate in the first translation process performed back-translation. Third, the first and second steps were repeated until they found no differences in meaning between the two versions. Finally, a medical nutrition professor who was not involved in the preceding steps discussed and compared back-translation text with the original text, including the expression of the translation and the choice of vocabulary and whether or not any terms needed to be modified due to clarity and cultural differences in translation.

### Sedentary time and stress level

Sedentary time was examined using the item, “How many hours a day do you usually spend sitting or lying down?” Subjective stress was investigated using the item, “How much stress do you usually feel?” The items were rated using a five-point Likert-type scale (“I feel very much”, “I feel a lot,”, “I feel a little,”, “I don’t feel very much and “I don’t feel much at all”).

### Instruments

#### The global physical activity questionnaire

The study evaluated physical activity using the Global Physical Activity Questionnaire (GPAQ) [20], which measures physical activity in daily life, including work and recreational activities. It comprises 16 items. Metabolic equivalents (METs) are used for analyzing the GPAQ data and expressing the intensity of physical activity. MET is the ratio of the working metabolic rate relative to the resting metabolic rate. 1 MET equals a caloric consumption of 1 kcal/kg/h. For the calculation of the total MET score, the study considered the intensity of physical activity, the total time spent on physical activity during a typical week as well as the number of days [21].

#### Energy intake assessment (food records)

Energy intake was obtained through a three-day food record, which was completed by the participants on two weekdays and one weekend day. They filled out the amount, recipe, and ingredients of food and snack consumed.

#### Nutrition quotients

The nutritional index was evaluated using the Nutrition Quotients (NQ) for Korean Adolescents. It is a tool for comprehensively evaluating the dietary quality and food behavior of middle- and high-school students [22]. It consists of five factors, namely, balance, regularity, diversity, practice, and environment, with a total of 19 items. The NQ score was calculated by multiplying the score calculated for each evaluation item by the weight of each evaluation item and summing the resultant values. The score for each factor of NQ was calculated by multiplying the evaluation item score in the factor by the item weight in the factor and adding them up. The score of the five factors and the NQ total were calculated as 0 to 100. The higher the score, the better the nutritional index.

#### Dutch eating behavior questionnaire for children (DEBQ-C)

The DEBQ-C is composed of 20 items and evaluates three types of eating behavior in children aged 7–12 years [10]. The first is emotional eating and consists of seven items about negative emotions, such as “If you feel depressed, do you get a desire for food?” The second type is external eating (six items) and asks whether or not food consumption is triggered by external circumstances such as “Do you feel like eating whenever you see or smell good food?” Lastly, restrained eating (seven items) pertains to the conscious restriction of food intake to maintain or control weight such as “Have you ever tried to avoid eating after your evening meal to lose weight?” The items were rated using a three-point Likert-type scale (1 = *no*, 2 = *sometimes*, 3 = *yes*) in which the scores range from is 1 to 3. High total scores for each eating behavior indicate greater endorsement of the behavior [10]. Previous research has confirmed the validity and reliability of the DEBQ-C in measuring the three targeted eating behaviors in studies in Taiwan [16], Spain [17], and Japan [18].

#### Yale food addiction scale for children

The Yale Food Addiction Scale for Children (YFAS-C) is the adolescent version of the Food Addiction Scale and measures eating addiction behaviors toward salty, fatty, and sweet food. The seven symptoms of substance dependence were revised to apply to eating behaviors based on the substance dependence criteria of the Diagnostic and Statistical Manual of Mental Disorders (DSM-IV) [23]. It has 25 items, which consist of an 18-item continuous scale (0 = *not at all*, 1 = *once a month*, 2 = *2–4 times per month*, 3 = *2–3 times per week*, 4 = *more than four times per week* or *every day*) and a 7-item dichotomous scale (0 = *no*, 1 = *yes*). The 22 items that comprise the continuous and dichotomous scales were converted into dichotomous data for seven symptoms of food addiction and clinical impacts of specific foods using the structured conversion coding sheet presented by the original author. Three items serve as primers, which are not reflected in the score. The degree of food addiction symptomatology is calculated by the number of symptoms in the range of 0–7 and is classified into two groups names *food addiction risk* and *food addiction*. This classification was dependent on whether or not specific symptoms of clinical impacts were included. If three or more symptoms of food addiction symptoms and clinical impacts were observed, then the respondent was classified as being in the food addiction group; if without clinical impacts from food addiction, the respondent was classified as being in a food addiction risk group [24].

The diagnosis of food addiction is frequently reported in individuals with bulimia nervosa or binge eating behaviors who also have a craving for food [25]. Food addiction is also associate with a high BMI [26–28] and psychological symptoms such as depression [29]. Additionally, the level of food addiction risk or number of food addiction symptoms are associated with eating behaviors such as binge eating and dietary restriction [23, 30–32]. Therefore, the YFAS-C is similar to DEBQ-C in that it measures problematic eating behaviors; hence, it was used to confirm convergent validity.

### Data analysis

Data analysis was conducted using SPSS Windows version 25.0 and JAMOVI version 1.6.23. The categories of weight status were overweight, obese, and morbidly obese. The study analyzed the differences between weight status classification and categorical variables using the chi-squared test. Group differences in continuous variables were analyzed using Welch’s analysis of variance (ANOVA). Welch’s ANOVA was used, because the sample size of the subjects was small, and the difference in the number of samples by group was large. The study examined the differences between the weight status groups using the Games–Howell post hoc test.

The study established factor structure and goodness-of-fit via confirmatory factor analysis (CFA), which was also performed using JAMOVI version 1.6.23 to verify that the results corresponded to the original three-factor model. With an assumption of normality, we used the maximum likelihood estimation. The association between the change in the dependent and independent variables was confirmed through a standardized coefficient that indicates the correlation between changes in independent variables and changes in dependent variables after CFA analysis. To test the suitability of the model, the study calculated for the chi-square test (χ^2^), the ratio of the chi-square value to degrees of freedom (χ^2^/*df*), composite fit index (CFI), Tucker–Lewis index (TLI), and standardized root mean square residual (SRMR). The resulting values (*χ*^2^/*df* < 2.0, RMSEA < 0.06, SRMR < 0.08 [33], CFI > 0.90, and TLI > 0.90 [34]) suggested adequate model fit.

The internal consistency of the overall DEBQ-C score and its individual subscales was evaluated using Cronbach’s alpha. The criterion for a suitable reliability value is > 0.70 [35]. Convergent validity was confirmed by examining the correlations between the scores of the DEBQ-C scores and those reflecting the number of symptoms of food addiction endorsed by the YFAS- C. Correlation coefficients of 0.1, 0.3, and 0.5 indicate small, moderate, and large correlations, respectively [36].

The study performed multinomial logistic regression analysis to confirm the association between scores for the three eating behaviors and weight status. Age and sex were corrected, because the DEBQ-C scores varied with age and sex. Similarly, we adjusted for the risk factors for obesity such as MET score, sedentary time, stress level, and NQ score.

## Results

### Participant characteristics

Twenty-eight (12%) of the 233 participants were overweight, 131 (56.2%) were obese, and 74 (30.9%) were morbidly obese. The average BMIs and *z*-scores of the three groups were 23.39 ± 1.31 (*z* = 1.4 ± 0.2), 26.85 ± 1.85 (*z* = 2.4 ± 0.3), and 32.50 ± 3.48 (*z* = 3.6 ± 0.6), respectively. The number of food addiction symptoms was significantly higher for participants in the morbidly obese group compared with to those in r the overweight or obese groups (Table 1).

**Table 1:** General characteristics according to obesity status in children and adolescents with high weight

Variables	Total (N = 233)	Overweight (N = 28)	Obese (N = 131)	Morbidly obese (N = 74)	*p* value
Sex (n, %)					
Boys	161 (69.10)	17 (60.71)	93 (70.99)	51 (68.92)	0.565
Girls	72 (30.90)	11 (39.29)	38 (29.01)	23 (31.08)	
Age (year)	11.4 ± 1.6	11.2 ± 1.5	11.2 ± 1.5	11.7 ± 1.8	0.160
Children (n, %)	139 (59.66)	17 (60.71)	84 (64.12)	37 (50.00)	0.122
Adolescents (n, %)	94 (40.34)	11 (39.29)	47 (35.88)	37 (50.00)	
Anthropometrics					
Height (cm)	**156.34 ± 10.47**	**152.31 ± 9.97**^**c**^	**155.09 ± 10.28**^**bc**^	**160.1 ± 10.04**^**a**^	**< 0.001**
Weight (kg)	**69.98 ± 17.05**	**54.74 ± 9.48**^**c**^	**65.18 ± 12.16**^**b**^	**84.24 ± 17.09**^**a**^	**< 0.001**
BMI (kg/m^2^)	**28.23 ± 3.95**	**23.39 ± 1.31**^**c**^	**26.85 ± 1.85**^**b**^	**32.50 ± 3.48**^**a**^	**< 0.001**
BMI z-score	**2.65 ± 0.85**	**1.41 ± 0.18**^**c**^	**2.35 ± 0.34**^**b**^	**3.63 ± 0.60**^**a**^	**< 0.001**
Waist (cm)	**86.68 ± 11.28**	**74.12 ± 7.55**^**c**^	**83.91 ± 7.59**^**b**^	**96.35 ± 10.58**^**a**^	**< 0.001**
Hip (cm)	**98.43 ± 9.58**	**90.18 ± 5.62**^**a**^	**95.59 ± 6.70**^**b**^	**106.57 ± 9.65**^**c**^	**< 0.001**
WHR	**0.88 ± 0.06**	**0.82 ± 0.06**^**c**^	**0.88 ± 0.06**^**b**^	**0.90 ± 0.06**^**a**^	**< 0.001**
Physical activity					
METs score	4077.12 ± 5694.53	3402.31 ± 3449.47	4717.39 ± 6703.92	3191.44 ± 4137.78	0.130
Sedentary time (hours/d)	6.85 ± 4.30	7.25 ± 3.38	6.82 ± 4.23	6.39 ± 4.82	0.141
NQ total score (0–100)	**58.33 ± 10.83**	**58.05 ± 2.01**^**ab**^	**59.89 ± 0.93**^**a**^	**55.68 ± 1.24**^**b**^	**0.028**
Balance (100)	53.33 ± 16.68	53.94 ± 3.15	54.64 ± 1.46	50.78 ± 1.95	0.283
Regularity (100)	61.62 ± 17.89	63.06 ± 3.39	62.23 ± 1.57	59.99 ± 2.10	0.629
Diversity (100)	**54.59 ± 20.11**	**49.95 ± 3.73**^**ab**^	**58.47 ± 1.73**^**a**^	**49.48 ± 2.30**^**b**^	**0.004**
Practice (100)	53.00 ± 18.30	53.69 ± 3.49	53.63 ± 1.61	51.64 ± 2.16	0.746
Environment (100)	72.00 ± 19.76	70.45 ± 3.69	74.66 ± 1.71	67.87 ± 2.28	0.055
Stress level (n, %)					
Very much	15 (6.44)	0 (0.00)	11 (8.40)	4 (5.41)	0.143
Somewhat	55 (23.61)	8 (28.57)	24 (18.32)	23 (31.08)	
Neutral	92 (39.48)	11 (39.29)	49 (37.40	32 (43.24)	
Not much	64 (27.47)	9 (32.14)	41 (31.30)	14 (18.92)	
Not at all	7 (3.00)	0 (0.00)	6 (4.58)	1 (1.35)	
Energy intake (kcal/d)	2055.05 ± 479.77	2073.89 ± 468.28	2029.32 ± 419.29	2093.47 ± 578.39	0.674
YFAS-C					
Food addiction^A^ (n, %)	17 (7.30)	2 (7.14)	8 (6.11)	7 (9.46)	0.675
Food addiction risk^B^ (n, %)	**29 (12.44)**	**1 (3.57)**	**13 (9.92)**	**15 (20.27)**	**0.031**
Food addiction symptoms ≥ 3^C^ (n, %)	**46 (19.74)**	**3 (10.71)**	**21 (16.03)**	**22 (29.73)**	**0.027**
Food addiction symptoms ^D^	**1.59 ± 1.51**	**1.21 ± 1.32**^**c**^	**1.37 ± 1.33**^**bc**^	**2.12 ± 1.76**^**a**^	**0.004**

Values below* p*-value < 0.5 are indicated in bold

Values are expressed as mean ± SD for continuous variables or as n (%) for categorical variables

*p* values for difference between groups from chi-squared test (categorical variables) or Welch’s analysis of variance (continuous variables)

Significantly different among the group were indicated by superscripts through the games-Howell post-hoc tests

BMI, Body Mass Index; WHR, Waist-Hip Ratio; NQ, Nutrition Quotient; YFAS-C, Yale Food Addiction Scale for Children

Overweight (BMI 85th-95th percentile), Obese (BMI ≥ 95th percentile) Morbidly Obese (BMI ≥ 120% of the 95th percentile) (the 2017 Korean National Growth Charts for children and adolescents)

Children (Age: 9–11 years old), Adolescents (Age: 12–18 years old)

^A^The number of food addiction symptoms is three or more and does not include "clinically significant impairment or distress by a specific food”

^B^The number of food addiction symptoms is three or more and include "clinically significant impairment or distress by a specific food”

^C^Among the 7 food addiction symptoms, three or more are classified in the food addiction group or the food addiction risk group

^D^The total number of food addiction symptoms is 7

### Validation

#### Factor structure

**Fig. 1:**
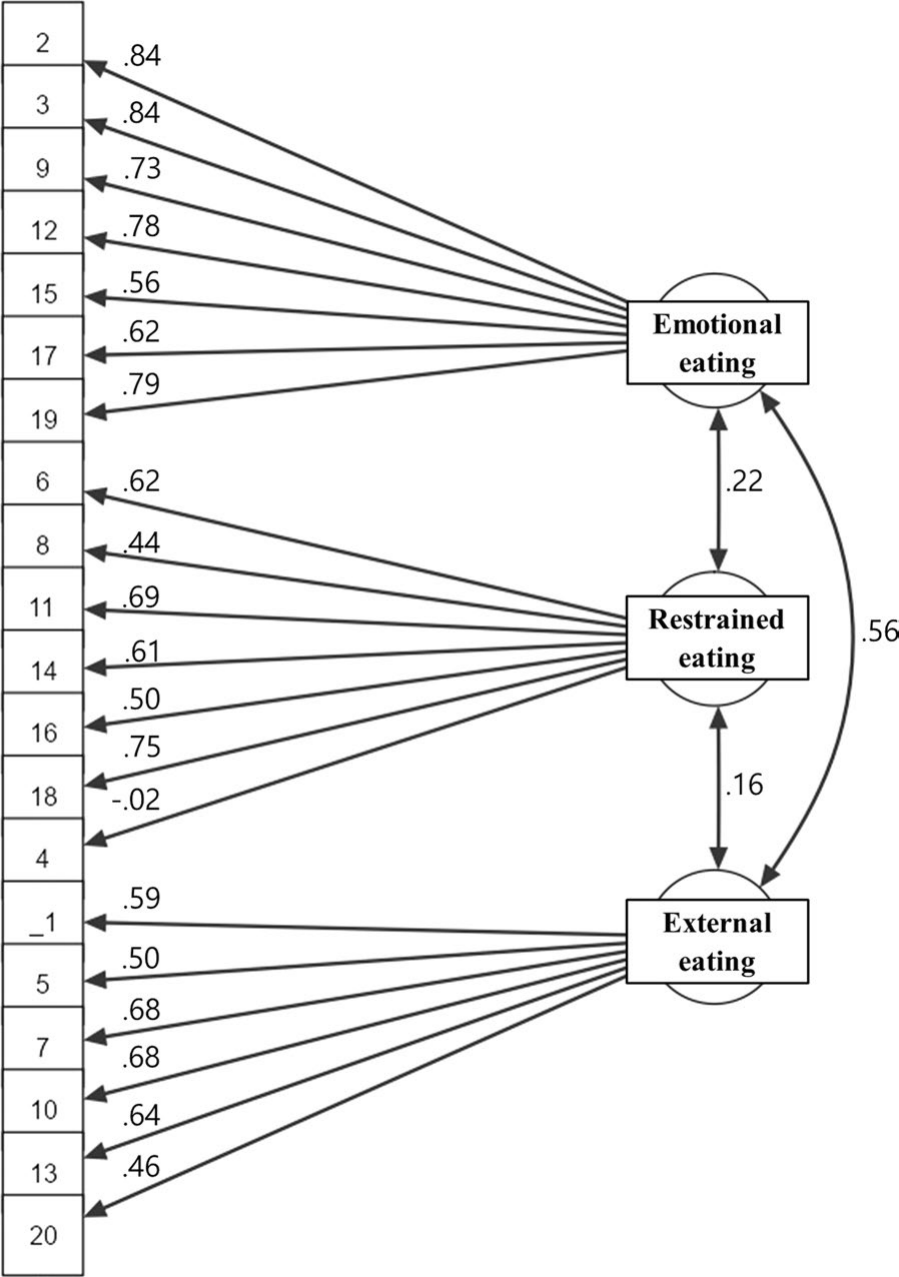
Confirmatory factor analysis of the DEBQ-C of children and adolescents with high weight

The study performed CFA to confirm that the three-factor model of the Korean version of DEBQ-C was consistent with the three-factor model of the original version. The three-factor model demonstrated adequate fit for DEBQ-C with 20 items (χ^2^ = 253, *df* = 167, *p* < 0.001, CFI = 0.944; TLI = 0.937; RMSEA = 0.047). Figure 1 presents the standardized coefficient estimates for the model. In the three-factor structure, seven items under emotional eating produced coefficients ranging from 0.56 to 0.84, while restrained eating has seven items with loadings ranging from − 0.02 to 0.75. Lastly, six items under external eating exhibited loadings from 0.46 to 0.68. Item 4 under restrained eating displayed a negative value (− 0.02).

#### Internal consistency

Internal consistency was evaluated using Cronbach’s alpha (Table 2). The three factors exhibited alpha values > 0.7 (Cronbach’s alpha = 0.707–0.890) [34]. Cronbach’s alpha for restrained eating reached 0.707 but increased to 0.769 after the exclusion of item 4, which, along with other items under this subscale, displayed a negative correlation (item–total subscale correlation =  − 0.027).

**Table 2:** Reliability estimates for each DEBQ-C subscale item of children and adolescents with high weight

	DEBQ-C items	Item-total subscale correlation	Cronbach’s alpha
Total items			0.943
Emotional eating	V2	0.764	0.890
	V3	0.778	
	V9	0.684	
	V12	0.744	
	V15	0.528	
	V17	0.589	
	V19	0.750	
Restrained eating	V4	-0.027	0.707
	V6	0.513	
	V8	0.381	
	V11	0.574	
	V14	0.477	
	V16	0.446	
	V18	0.605	
External eating	V1	0.513	0.757
	V5	0.417	
	V7	0.580	
	V10	0.554	
	V13	0.545	
	V20	0.397	

Reliability analysis was performed through Cronbach’s alpha

Criteria for suitable reliability values: Cronbach's Alpha > .70

#### Convergent validity

To confirm convergent validity, the study examined the correlations between the number of symptoms of food addiction according to the YFAS-C and scores on the three types of eating behavior in DEBQ-C. The number of symptoms of food addiction was correlated with emotional eating (*r* = 0.440, *p* < 0.001) and external eating (*r* = 0.429, *p* < 0.001). The correlation between number of symptoms of food addiction and restrained eating was not significant.

### Overeating in relation to weight status

#### DEBQ-C subscale scores

Table 3 presents the scores for the DEBQ-C subscale according to weight status. *p*-Values for difference between groups were derived from Welch’s ANOVA. Among children, the tendency toward emotional eating was higher for the obese (1.38 ± 0.52) than for the overweight (1.15 ± 0.22; *F* = 4.655, *p* = 0.013) group. The tendency toward external eating was higher for the obese (2.09 ± 0.47) than for the overweight (1.83 ± 0.35; *F* = 3.361, *p* = 0.043) group. The study observed no significant difference in restrained eating according to weight status among children and adolescents.

**Table 3:** Mean of the DEBQ-C subscale according to obesity status of children and adolescents with high weight

	Total				
	Total (N = 233)	Overweight (N = 28)	Obese (N = 131)	Morbidly obese (N = 74)	*p* value
Emotional eating^A^	1.37 ± 0.49	1.24 ± 0.38	1.39 ± 0.52	1.37 ± 0.48	0.211
Restrained eating^B^	1.92 ± 0.38	1.94 ± 0.36	1.93 ± 0.39	1.90 ± 0.39	0.848
External eating^C^	2.01 ± 0.45	1.92 ± 0.46	2.05 ± 0.47	1.98 ± 0.42	0.314

	Children				
	Total (N = 139)	Overweight (N = 18)	Obese (N = 84)	Morbidly obese (N = 37)	*p* value
Emotional eating	1.33 ± 0.47	1.15 ± 0.22^b^	1.38 ± 0.52^a^	1.32 ± 0.043^ab^	0.013
Restrained eating	1.89 ± 0.37	1.94 ± 0.40	1.91 ± 0.36	1.83 ± 0.38	0.457
External eating	2.03 ± 0.45	1.83 ± 0.35^b^	2.09 ± 0.47^a^	2.01 ± 0.45^ab^	0.043

	Adolescents				
	Total (N = 94)	Overweight (N = 10)	Obese (N = 47)	Morbidly obese (N = 37)	*p* value
Emotional eating	1.42 ± 0.51	1.41 ± 0.55	1.42 ± 0.51	1.43 ± 0.53	0.997
Restrained eating	1.96 ± 0.40	1.93 ± 0.30	1.96 ± 0.44	1.97 ± 0.39	0.928
External eating	1.98 ± 0.44	2.08 ± 0.59	1.99 ± 0.46	1.95 ± 0.38	0.771

Values are expressed as mean ± SD

*p* values for difference between groups from welch’s analysis of variance (continuous variables)

Significantly different among the group were indicated by superscripts through the games-howell post-hoc tests

^A^ It means overeating due to negative emotions, and the higher the score, the higher the emotional eating. 7 questions. Consists of 1 (no) to 3 (yes) points

^B^ It refers to overeating when the urge to eat is suppressed or the cognitive effort to eat less is released. The higher the score, the higher the degree of restrained eating. 7 questions. Consists of 1 (no) to 3 (yes) points

^C^ It means that eating is induced impulsively when seeing or smelling food regardless of the state of internal psychological arousal. The higher the score, the higher the degree of external eating. 6 questions. Consists of 1 (no) to 3 (yes) points

Among adolescents, the tendency toward restrained eating was higher for girls (2.14 ± 0.36) than for boys (1.89 ± 0.40; *F* = 8.80, *p* = 0.004). The study noted no significant difference between boys and girls in children (emotional eating: boys = 1.30 ± 0.46, girls = 1.40 ± 0.50, *F* = 1.2752, *p* = 0.246; restrained eating: boys = 1.91 ± 0.38, girls = 1.85 ± 0.35, *F* = 0.8589, *p* = 0.373; external eating: boys = 2.03 ± 0.47, girls = 2.05 ± 0.43, *F* = 0.0467, *p* = 0.137). In addition, the study found no significant difference between children and adolescents (emotional eating: children = 1.33 ± 0.47, adolescents = 1.42 ± 0.51, *F* = 1.838, *p* = 0.177; restrained eating: children = 1.89 ± 0.37, adolescents = 1.96 ± 0.40, *F* = 1799, *p* = 0.181; external eating: children = 2.03 ± 0.45, adolescents = 1.98 ± 0.44, *F* = 0.685, *p* = 0.409).

#### Relationship between the three eating behaviors and weight status

The study performed multinomial logistic regression analysis to confirm the association between the three eating behaviors of the DEBQ-C and weight status (Table 4). Analyses were adjusted by variables related to the risk factors of obesity, such as MET scores, age, sex, sedentary time, NQ score, and stress level. Emotional eating was positively associated with obese status (OR: 2.008; 95% CI 1.973–2.043) and morbidly obese status (OR: 1.580; 95% CI 1.559–1.601), while external eating was positively associated with obese status (OR: 2.074; 95% CI 2.029–2.119). Conversely, restrained eating was negatively associated with obese status (OR: 0.373; 95% CI 0.368–0.379) and morbidly obese status (OR: 0.480; 95% CI 0.471–0.489).

**Table 4:** Association between DEBQ-C subscales and obesity status in children and adolescents with high weight

	Emotional eating		Restrained eating		External eating	
	OR	95% CI	OR	95% CI	OR	95% CI
Overweight	1.000 (ref)	1.000 (ref)	1.000 (ref)			
Obese	**2.008**	**(1.973–2.043)**	**0.373**	**(0.368–0.379)**	**2.074**	**(2.029–2.119)**
Morbidly obese	**1.580**	**(1.559–1.601)**	**0.480**	**(0.471–0.489)**	1.001	(0.982–1.020)

Values below* p*-value < 0.5 are indicated in bold

Multinomial logistic regression analysis between DEBQ-C subscales and obesity status adjusted for age, sex, METs score, Sedentary time, NQ total scores, stress level

*OR* odds ratio, *CI* confidence interval

## Discussion

This study evaluated the psychometric properties of the Korean version of the DEBQ-C. In summary, the findings lend support to the validity of this version and suggest that the translation is suitable for use in studies that examine overeating behavior among Korean children and adolescents with a high weight.

The three-factor model of the 20 items displayed adequate goodness-of-fit, and the measure exhibited good internal consistency and convergent validity with the symptoms of food addiction and weight status.

### Factor structure and item refinement issues

CFA revealed that the DEBQ-C had a satisfactory three-factor structure. However, in the case of item 4, the standardized coefficient estimates produced a negative value (− 0.02). The item–total subscale correlation was also − 0.027, which was less than the recommended removal range of 0.30 [37]. Other studies that verified the effectiveness of the DEBQ-C also reported that item 4 displayed similar patterns such as loading on a different factor [16, 38–40]. Based on the present and others’ findings, the recommendation to exclude item 4 should be revisited. When it is included we suggest it should be modified by providing additional context such as revising the sentence to convey restricted eating for the purpose of health and weight management.

### Convergent validity of the DEBQ-C

In summary, the correlation between DEBQ-C and symptoms of food addiction measured by the YFAS-C were adequate, and convergent validity was further verified with association between the DEBQ-C and weight status. Whilst food addiction and the three eating behaviors of the DEBQ-C exhibit a common feature that is, constant craving for food, but differences were found in associations according to the type of eating behavior. The current study found similar results to others [30, 41, 42] in that emotional and external eating correlated highly with food addiction, which is the constant desire for food and binge eating. In addition, in terms of the three eating behaviors, the levels of emotional and external eating were higher for the obese and morbidly obese participants than for those in the overweight group. Furthermore, they were found to be correlated with the BMI *z*-score. in sum, these results provide additional evidence for the convergent validity of the DEBQ-C and confirmed associations between problematic eating behavior and high weight status.

In contrast to the present findings, previous studies have reported high weight to be associated with an increased tendency to engage in restrained eating among children [16, 17, 38]. The current study also found that girls displayed a higher degree of restrained eating than boys. This may be because adolescent girls have been reported to be more concerned about appearance and body dissatisfaction than boys [43].

### Limitations

This study has several limitations: First, it was unable to confirm the stability of the DEBQ-C factor structure in normal-weight Korean children and adolescents, because the recruited participants were overweight. Similarly, we were unable to test the discriminant validity of the scale. For the generalized use of the DEBQ-C, future studies that intend to target normal body weight, children with nonclinical obesity, and larger areas are needed. Second, this study is cross-sectional and test–retest reliability was not measured. Third, the study was limited in assessment of eating disorder diagnostic and other features.

Conversely, this study has strengths. These include the use of validated measures and adequate sample size to detect differences.

## Conclusion

This study reproduced the stable factor structure of the Korean version of the DEBQ-C and provided support for its validity when used in Korean children and adolescents with a high weight. In conclusion, the DEBQ-C is a useful tool for evaluating the overeating behaviors of children and adolescents in Korea. For the wider use of DEBQ-C, further studies involving other samples are necessary.

## Data Availability

The datasets used and/or analysed during the current study are available from the corresponding author on reasonable request.
